# Emergence of ceftazidime-avibactam resistance mediated by KPC variants KPC-71 and KPC-78 in ST463 *Pseudomonas aeruginosa*

**DOI:** 10.1128/spectrum.01095-26

**Published:** 2026-07-10

**Authors:** Fengjiao Yang, Yingbo Sun, Wenxia Chen, Dandan Shen, Jichao Zhu, Zijiu Sun, Yufei Cai, Yue Zhang, Hui Shen, Fenyong Sun, Yanghua Xiao, Cizhong Jiang, Zhongqi Cui

**Affiliations:** 1 Department of Clinical Laboratory, Shanghai Tenth People’s Hospital, School of Medicine, Tongji University 481875, Shanghai, China; 2 Department of Central Laboratory, Clinical Medicine Scientific and Technical Innovation Park, Shanghai Tenth People’s Hospital, Shanghai, China; 3 Shanghai Key Laboratory of Pathogenic Fungi Medical Testing, Shanghai Pudong New Area People’s Hospital, Shanghai Jiao Tong University School of Medicine 56694, Shanghai, China; 4 Department of Clinical Laboratory, Shanghai Tenth People’s Hospital, School of Life Sciences and Technology, Tongji University 598788, Shanghai, China; 5 Fifth School of Clinical Medicine of Zhejiang Chinese Medical University and Affiliated Central Hospital of Huzhou University, Huzhou Central Hospital 235957, Huzhou, China; 6 Baishan Annie Wright Schools 634046 , Qingdao, Shandong, China; 7 Department of Respiratory and Critical Care Medicine, The First Affiliated Hospital, Jiangxi Medical College, Nanchang University, Nanchang, Jiangxi, China; University of Dundee, Dundee, United Kingdom

**Keywords:** CRPA, KPC-71, KPC-78, ceftazidime-avibactam resistance, ST463

## Abstract

*Pseudomonas aeruginosa* is a well-recognized opportunistic pathogen and a leading cause of healthcare-associated infections. The shrinking effectiveness of available antimicrobial therapies has intensified the global threat posed by carbapenem-resistant *P. aeruginosa* (CRPA). Here, we elucidate the mechanisms of ceftazidime-avibactam (CZA) resistance mediated by the rare KPC variants, KPC-71 and KPC-78, identified during the treatment of CRPA infections. Two CZA-resistant *P. aeruginosa* strains, SY-206885 and HZ-231016032, were isolated from critically ill male patients with severe pneumonia. Whole-genome sequencing assigned both isolates to the high-risk sequence type 463 (ST463). Isolate SY-206885 harbors the *bla*_KPC-71_ gene, while HZ-231016032 carries *bla*_KPC-78_. Cloning and expression of these genes in *P. aeruginosa* PAO1 conferred a marked increase in the CZA minimum inhibitory concentration. Notably, expression of KPC-71 or KPC-78 conferred CZA resistance while simultaneously reducing carbapenem hydrolytic activity, a trade-off previously described for some KPC variants but still rarely documented in *P. aeruginosa*. Structural analysis and kinetic profiling showed that, relative to wild-type KPC-2, both KPC-71 and KPC-78 exhibited reduced catalytic turnover but increased substrate affinity for ceftazidime, together with significantly weakened binding to avibactam. In addition, elevated expression of MexAB-OprM and AmpC-related determinants in the clinical isolates likely further enhanced the high-level CZA resistance phenotype. These findings highlight the capacity of the ST463 CRPA lineage to evolve CZA resistance through KPC structural diversification under antimicrobial pressure and underscore the need for close surveillance during therapy.

**IMPORTANCE:**

In this study, we report the detection of the uncommon KPC variants KPC-71 and KPC-78 in clinical sequence type 463 (ST463) carbapenem-resistant *Pseudomonas aeruginosa* isolates exhibiting resistance to ceftazidime-avibactam (CZA). We demonstrate that CZA resistance is driven by specific structural alterations—a serine insertion between residues 182 and 183 or a D179A substitution within the Ω-loop—that reshape the functional balance of the KPC enzyme. These changes appear to create an evolutionary trade-off by improving ceftazidime recognition while weakening avibactam-mediated inhibition. Given the widespread dissemination of the ST463 lineage in China, the emergence of these variants highlights the urgent need for clinicians to monitor for CZA resistance development during therapy.

**CLINICAL TRIALS:**

This study is registered with ClinicalTrials.gov as ChiCTR2500105846.

## INTRODUCTION

*Pseudomonas aeruginosa* is an important opportunistic pathogen that is frequently associated with severe hospital-acquired infections, particularly in critically ill and immunocompromised patients (1, 2). Its remarkable genomic plasticity enables rapid adaptation to antimicrobial pressure. This adaptability, together with its intrinsic resistance and ability to acquire additional resistance determinants, has made the treatment of *P. aeruginosa* infections increasingly challenging. Since the first report of KPC-producing *P. aeruginosa* in 2007, carbapenem-resistant *P. aeruginosa* (CRPA) has spread globally (3). While lineages such as sequence type 235 (ST235), ST111, and ST233 are recognized as major epidemic clones worldwide, ST463 has emerged as a predominant high-risk clone in China and is increasingly associated with carbapenem resistance and adverse clinical outcomes (4, 5). The success of this lineage has raised growing concern because of its capacity to accumulate multiple resistance mechanisms and persist in clinical environments.

In *P. aeruginosa*, resistance to β-lactams and carbapenems is multifactorial. Common mechanisms include the loss or downregulation of outer membrane proteins, particularly OprD, which limits carbapenem entry (6), as well as the upregulation of multidrug efflux systems such as MexAB-OprM and MexEF-OprN, which reduce intracellular antibiotic accumulation (7). In addition, some isolates acquire carbapenemases, a class of β-lactamases capable of hydrolyzing carbapenems (8). Among these enzymes, KPC-type serine carbapenemases are of particular concern, and KPC-2 has become one of the dominant carbapenem resistance determinants in CRPA isolates in China (9).

Ceftazidime-avibactam (CZA), a combination of a third-generation cephalosporin and a non-β-lactam β-lactamase inhibitor, has expanded therapeutic options for infections caused by organisms producing class A, class C, and some class D β-lactamases, including KPC enzymes (5, 10, 11). Consequently, CZA has been increasingly used to treat infections caused by carbapenem-resistant gram-negative pathogens. However, the clinical introduction of CZA has been accompanied by a growing number of reports describing the emergence of resistance during therapy (12, 13). In *P. aeruginosa*, CZA resistance may result from multiple mechanisms, including metallo-β-lactamase production, altered permeability, efflux pump overexpression, and structural variation in β-lactamases (14–19). Among these mechanisms, mutations in KPC enzymes are particularly important because they may directly alter substrate recognition and inhibitor binding. Accumulating evidence from Enterobacterales and, less commonly, from *P. aeruginosa* suggests that substitutions or insertions in critical structural regions of KPC, particularly in the Ω-loop, can reduce susceptibility to avibactam while modifying β-lactam hydrolysis profiles (20, 21). These adaptive changes are clinically important because they may arise under antibiotic selection pressure and complicate both phenotypic detection and therapeutic decision-making.

In this study, we characterized two CZA-resistant clinical ST463 *P. aeruginosa* isolates carrying *bla*_KPC-71_ or *bla*_KPC-78_. Through genomic, microbiological, biochemical, and structural analyses, we sought to clarify the contribution of these rare KPC variants to CZA resistance and to define the additional resistance-associated features present in these clinical isolates.

## RESULTS

### Clinical strain characterization

**Fig 1:**
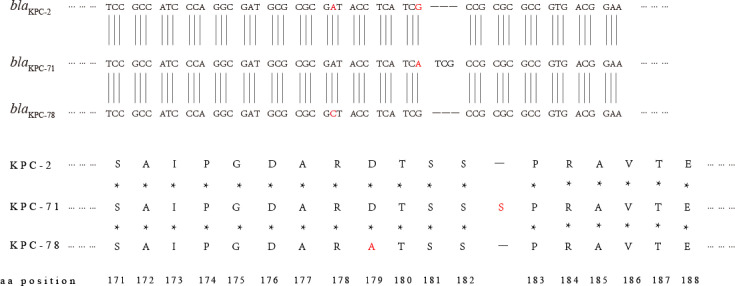
Amplicon alignments between *bla*_KPC-2_ and *bla*_KPC-71_, *bla*_KPC-78_ in nucleotide and amino acid (aa) sequences surrounding the mutation. One mutated nucleotide and an insertion of three nucleotides were identified in the *bla*_KPC-71_ gene compared to bla_KPC-2_, which led to serine between amino acid sequence positions 182 and 183 of the *bla*_KPC-2_ protein. One mutated nucleotide was identified at the *bla*_KPC-78_ gene compared to *bla*_KPC-2_, which resulted in the substitution of aspartic acid with alanine at amino acid sequence position 179. The red letters represent inconsistent bases and amino acids. Dotted line, common sequence; broken line, insertion of three nucleotides; *, common amino acid; boldface font, insertion of an amino acid. A, Ala; D, Asp; E, Glu; G, Gly; I, Ile; P, Pro; R, Arg; S, Ser; T, Thr; V, Val; Y, Tyr.

**Fig 2:**
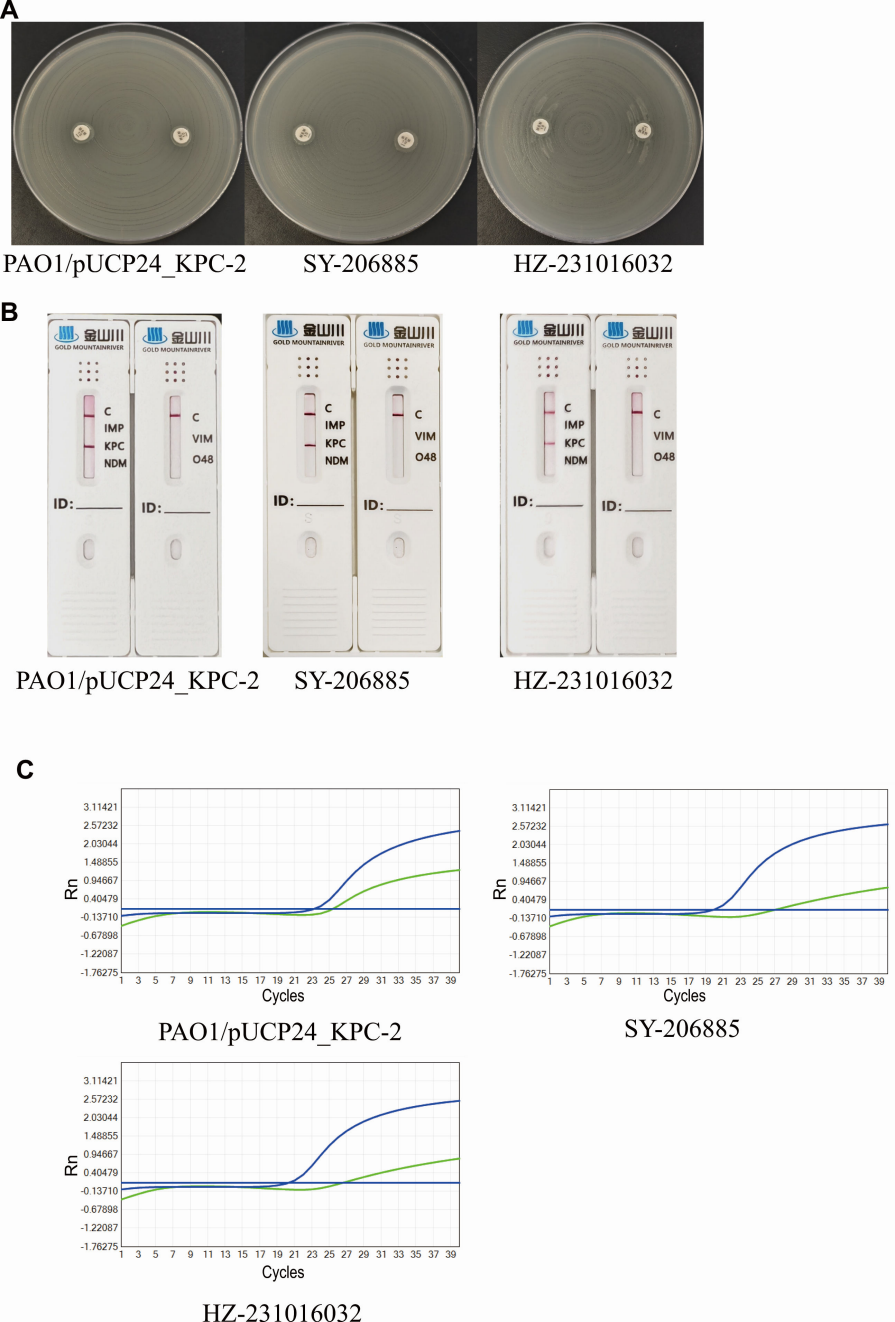
Phenotypic and molecular detection of KPC-71 and KPC-78. (**A**) Modified carbapenem inactivation method showing the inhibition zones produced by the indicator strain. A reduced or absent inhibition zone indicates carbapenem inactivation by the test isolate and is interpreted as a positive result for carbapenemase production. (**B**) NG-Test Carba for detecting KPC-71 and KPC-78. The appearance of both the control line (**C**) and the KPC line indicates a positive result for KPC carbapenemase, whereas no signal was observed for VIM, NDM, IMP, or OXA-48. (**C**) qPCR amplification curves for *bla*_KPC_ detection. Positive amplification curves were observed in PAO1/pUCP24_KPC-2, SY-206885, and HZ-231016032, confirming the presence of *bla*_KPC_ variants in these strains.

Two clinical *P. aeruginosa* strains, SY-206885 and HZ-231016032, were isolated from sputum samples of critically ill male patients with severe pneumonia. The patient infected with SY-206885 achieved infection control following treatment with a ceftazidime-avibactam-based regimen. The second patient, infected with HZ-231016032, was successfully treated with colistin, followed by cefepime, amikacin, and CZA. Both strains exhibited resistance to fluoroquinolones, β-lactams, and β-lactam/β-lactamase inhibitor combinations, while remaining susceptible to colistin and amikacin. Based on these profiles, the strains were classified as difficult-to-treat resistant *P. aeruginosa* (6, 22). Whole-genome sequencing identified both isolates as belonging to ST463, a lineage widely recognized as a high-risk clone in China because of its multidrug-resistant phenotype and epidemic potential (6). Both isolates harbored multiple resistance genes, including *catB7*, *fosA*, *bla*_PAO_, *aph(3′)-IIb*, and *bla*_OXA-486_. Notably, SY-206885 carried a mutant *bla*_KPC_ gene designated *bla*_KPC-71_, while HZ-231016032 carried *bla*_KPC-78_. As shown in Fig. 1, KPC-71 results from a three-nucleotide insertion, introducing a serine residue between amino acids 182 and 183, whereas KPC-78 contains a nucleotide substitution at position 531 that results in a D179A amino acid change. To evaluate diagnostic detectability, we employed the modified carbapenem inactivation method (mCIM), qPCR, and the NG-Test Carba 5 assay. Similar to KPC-2-producing strains, SY-206885 and HZ-231016032 tested positive in all assays (Fig. 2A through C), indicating that these variants remain detectable by routine carbapenemase detection methods.

### KPC-71- and KPC-78-carrying plasmids

**Fig 3:**
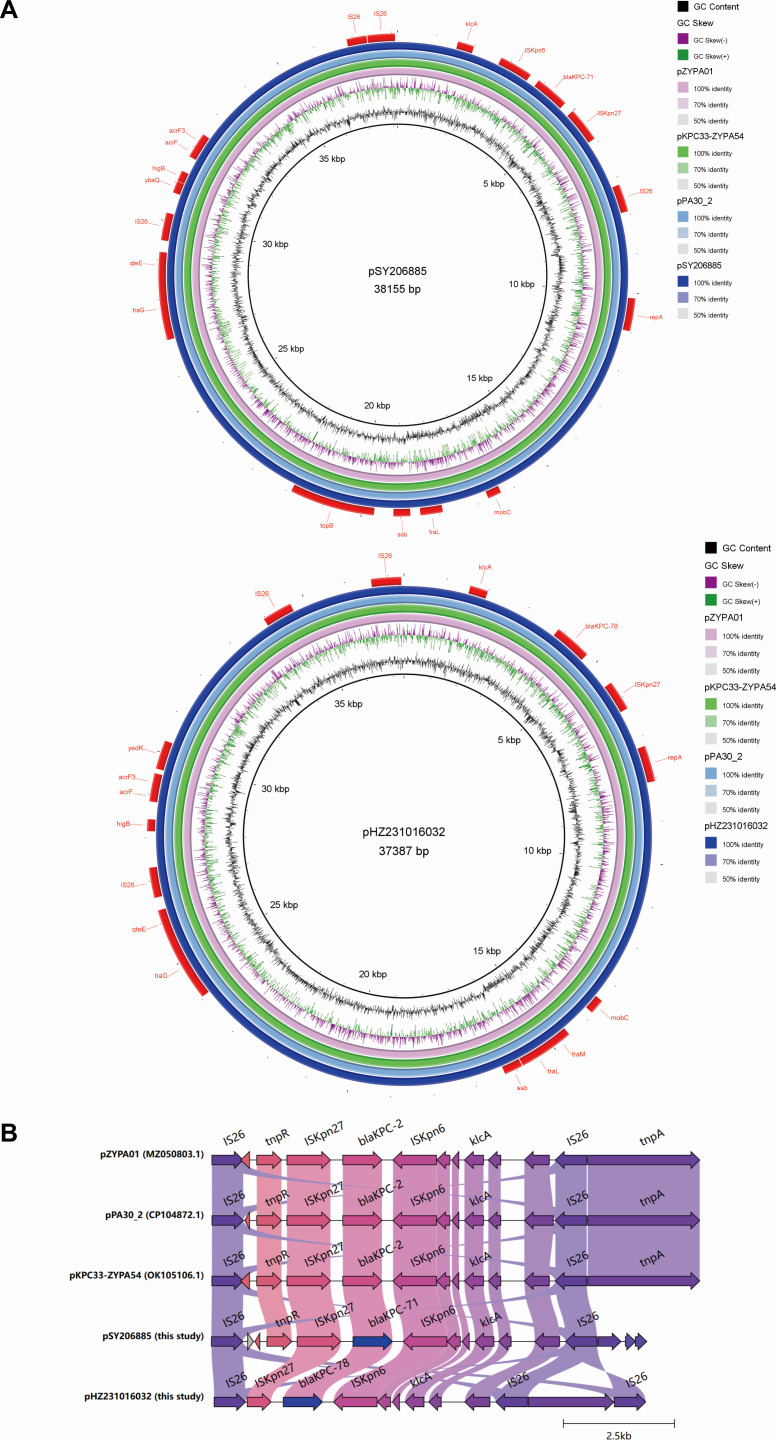
Characterization of *bla*_KPC-71_-harboring plasmid pSY206885 and *bla*_KPC-78_-harboring plasmid pHZ231016032. (**A**) Schematic map of plasmids pSY206885 and pHZ231016032. Sequence alignment of plasmids pSY206885 and pHZ231016032 with sequences of the plasmids pZYPAO1 (accession number: MZ050803), pPA30_2 (accession number: CP104872), and pKPC-33-ZYPA54 (accession number: OK105106). GC content is shown in the inner circle in black. (**B**) Comparison of the *bla*_KPC_ genetic context from pZYPAO1, pPA30_2, pKPC-33-ZYPA54, pSY206885, and pHZ231016032.

Long-read sequencing was further performed to obtain the complete sequences of the KPC-carrying plasmids. Plasmid analysis revealed that pSY-206885 was 38,155 bp in length with a G+C content of 58.9%, whereas pHZ-231016032 was 37,387 bp long with a G+C content of 58.65% (Fig. 3A). The mutant *bla*_KPC-71_ and *bla*_KPC-78_ genes were located in a conserved mobile genetic context, with ISKpn27 and IS26 upstream and IS26 downstream. Comparative sequence analysis showed that pSY-206885 shared 100% identity and 100% coverage with plasmids pZYPAO1 (GenBank: MZ050803), pZYPA54 (OK105106), and pPA30 (CP104872). Similarly, pHZ-231016032 was nearly identical to these reference plasmids (99.99% coverage and 100% identity). The mutant *bla*_KPC_ genes shared the same replication-associated genes and conserved genetic environment (IS26-ISKpn27-bla_KPC_-IS*Kpn6*) as the reference plasmids (Fig. 3B). Conjugation experiments were performed to assess plasmid transferability. Transconjugants were successfully obtained and verified by PCR. Compared with the recipient PAO1^RifR^, transconjugants PAO1^RifR^/pSY-206885 and PAO1^RifR^/pHZ-231016032 exhibited an approximately 256-fold increase in CZA minimum inhibitory concentrations (MICs) (Table 1), confirming plasmid-mediated transfer of the CZA resistance phenotype.

**TABLE 1:** Antibiotic susceptibility of the strains used in this study (mg/L)^*a*^

Strains	CZA	FEP	CAZ	PTZ	IPM	MEM	CIP	AMK	CST
SY-206885	1,024	64	>128	>128	64	128	>64	1	2
HZ-231016032	512	>128	>128	>128	64	64	>64	4	2
*P. aeruginosa* PAO1^RifR^	0.5	2	0.5	8	1	1	<0.125	2	<0.25
PAO1^RifR^/pSY-206885^*b*^	64	>64	>128	>128	≥16	≥16	>64	8	1
PAO1^RifR^/pHZ-231016032^*c*^	32	>64	128	>128	≥16	≥16	>64	16	2
PAO1/pUCP24^*d*^	0.5	1	0.25	4	0.5	1	<0.125	2	<0.25
PAO1/pUCP24_KPC-2^*e*^	4	>64	64	>32	≥64	≥64	0.125	1	0.25
PAO1/pUCP24_KPC-71*f*	32	>32	64	16	1	1	0.125	1	2
PAO1/pUCP24_KPC-78^*g*^	32	>32	>128	16	1	1	0.125	1	2
*P. aeruginosa* ATCC 27853	0.125	1	0.25	2	0.25	0.25	0.125	0.5	<0.25

^*a*^ Avibactam was added at 4 mg/L.

^*b*^ PAO1^RifR^/pSY-206885, *P. aeruginosa* PAO1^RIF^ was transformed by pSY-206885 plasmid carrying wild-type *bla*_KPC-71_ gene.

^*c*^ PAO1^RifR^/pHZ-231016032, *P. aeruginosa* PAO1^RIF^ was transformed by pHZ-231016032 plasmid carrying *bla*_KPC-78_ gene.

^*d*^ PAO1/pUCP24, *P. aeruginosa* was transformed by expression plasmid pUCP24 as a control.

^*e*^ PAO1/pUCP24_KPC-2, *P. aeruginosa* was transformed by a pKPC-2 plasmid carrying wild-type *bla*_KPC-2_ gene.

^*f*^ PAO1/pUCP24_KPC-71, *P. aeruginosa* was transformed by a pKPC-71 plasmid carrying *bla*_KPC-71_ gene.

^*g*^ PAO1/pUCP24_KPC-78, *P. aeruginosa* was transformed by a pKPC-78 plasmid carrying *bla*_KPC-71_ gene.

### KPC-71 and KPC-78 mediate CZA resistance

To confirm that *bla*_KPC-71_ and *bla*_KPC-78_ are responsible for the resistance phenotype, the genes were cloned and expressed in *P. aeruginosa* PAO1, and transformation was verified by PCR. PAO1 transformants carrying the pUCP24 vector and wild-type *bla*_KPC-2_ remained susceptible to CZA (MIC = 4 µg/mL), although the KPC-2 transformant was resistant to meropenem and imipenem (Table 1). In contrast, transformants expressing KPC-71 or KPC-78 exhibited CZA MICs of 32 μg/mL and, interestingly, regained susceptibility to meropenem and imipenem (1 μg/mL). This phenotype aligns with previous reports for *Escherichia coli* expressing KPC-71 (15) and other KPC variants (e.g., KPC-33, -41, -50, -83) that confer CZA resistance at the cost of carbapenemase activity (16–19). These results extend this resistance trade-off to *P. aeruginosa*, where such observations remain relatively uncommon, and indicate that KPC-71 and KPC-78 can confer CZA resistance in this species while simultaneously impairing carbapenem hydrolysis.

### Kinetic properties of KPC variants

To further investigate the mechanism of CZA resistance mediated by KPC-71 and KPC-78, enzymatic kinetics were analyzed to elucidate the biochemical basis of resistance. Compared to KPC-2, both KPC-71 and KPC-78 exhibited reduced catalytic efficiency (*K*_cat_/*K_m_*) for ceftazidime (a 2-fold and 1.25-fold reduction, respectively). However, they displayed a significantly higher affinity (lower *Km*) for ceftazidime, with approximately 3-fold and 1.89-fold lower *Km* values, respectively, than KPC-2 (Table 2).

**TABLE 2:** Kinetic parameters of purified β-lactamases KPC-2, KPC-71, and KPC-78^*a*^

	KPC-2				KPC-71				KPC-78			
	*K*_*m*_ (µM)	*K*_cat_ (s^-1^)	*K*_cat_/*K*_*m*_ (s^-1^ µM^-1^)	*K*_*i*_ (µM)	*K*_*m*_ (µM)	*K*_cat_ (s^-1^)	*K*_cat_/*K*_*m*_ (s^-1^ µM^-1^)	*K*_*i*_ (µM)	*K*_*m*_ (µM)	*K*_cat_ (s^-1^)	*K*_cat_/*K*_*m*_ (s^-1^ µM^-1^)	*K*_*i*_ (µM)
Meropenem	16.620	6.232	0.375	/	27.820	9.106	0.327 ↓	/	5.981	1.391	0.233 ↓	/
Imipenem	101.300	18.890	0.186	/	ND	ND	ND ↓	/	139.500	12.550	0.090 ↓	/
Ceftazidime	548.800	38.530	0.070	/	175.200	6.005	0.034 ↓	/	289.400	16.290	0.056 ↓	/
Nitrocefin	3.735	14.360	3.845	/	2.320	5.064	2.183 ↓	/	3.54	6.798	1.920 ↓	/
Avibactam	/	/	/	0.066	/	/	/	0.189 ↑	/	/	/	0.176 ↑

^*a*^ ND, not determined due to a low initial rate of hydrolysis. *K*_cat_, turnover; *K_m_*, Michaelis constant (affinity); *K*_cat_*/K*_*m*_, specificity constant (hydrolysis). “/” indicates that the value was not determined.

Conversely, the hydrolytic efficiency (*K*_cat_/*K_m_*) against carbapenem-associated substrates (including meropenem and imipenem) was markedly lower for the variants than for KPC-2; notably, KPC-71 showed almost no detectable hydrolysis of imipenem. This biochemical profile is consistent with the restored carbapenem susceptibility observed in the transformants during antimicrobial susceptibility testing. To assess inhibitor efficacy, *K_i_* values for avibactam were determined. Both KPC-71 and KPC-78 showed higher *K*_*i*_ values than KPC-2 (Table 2), indicating a diminished binding affinity of avibactam for the mutant enzymes.

To further evaluate the inhibitory activity of the enzyme inhibitor against KPC-2, KPC-71, and KPC-78, we determined the 50% inhibitory concentration (IC_50_) of avibactam, tazobactam, and clavulanic acid (Table 3). The IC_50_ value of avibactam against KPC-71 exhibited an approximately 15-fold higher than wild-type KPC-2 and about 11-fold higher against KPC-78 than against wild-type KPC-2, supporting the conclusion that both variants have substantially reduced susceptibility to avibactam inhibition. Furthermore, tazobactam and clavulanic acid against KPC-71 exhibited approximately 2-fold and 1.5-fold higher IC_50_ values, respectively, than those against KPC-2. Similarly, the IC_50_ against KPC-78 are about 3-fold and 2-fold higher, respectively. These data indicate that CZA resistance mediated by KPC-71 and KPC-78 is associated with an altered functional balance characterized by enhanced ceftazidime affinity, reduced catalytic turnover, and weakened avibactam inhibition.

**TABLE 3:** IC_50_ of β-lactamase inhibitors against KPC-2, KPC-71, and KPC-78^*a*^

Inhibitor	IC_50_ (µM)		
	KPC-2	KPC-71	KPC-78
Avibactam	0.027	0.408	0.295
Tazobactam	1.865	3.712	5.589
Clavulanic acid	1.013	1.521	2.031

^*a*^ IC_50_ represents the concentration of a drug that is required for 50% inhibition of the enzymatic activity.

### Overexpression of efflux pumps is associated with CZA resistance

**Fig 4:**
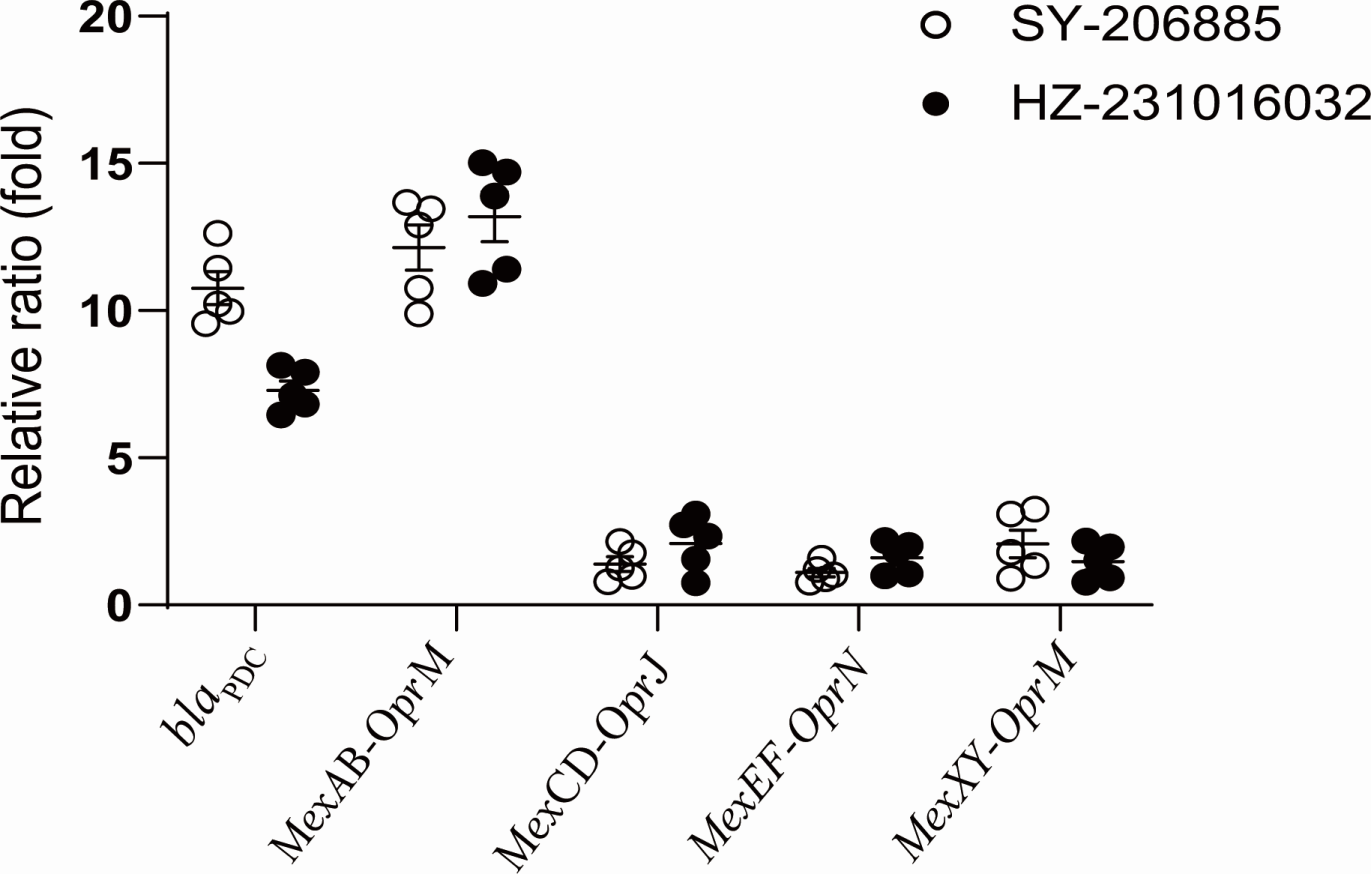
Relative ratios of gene expression of AmpC and efflux pumps. Gene expression was normalized to the *rpsL* housekeeping gene, and expression levels were indicated as a ratio to the expression level in *P. aeruginosa* strain PAO1.

In our study, *P. aeruginosa* SY-206885 and HZ-231016032 strains exhibited high-level resistance to CZA (MICs of 1,024 μg/mL and 512 μg/mL, respectively), but the transformants (PAO1/pUCP24_KPC-71 and PAO1/pUCP24_KPC-78) exhibited lower levels of resistance (MIC = 32 µg/mL for both). This discrepancy suggested that mechanisms in addition to KPC-71 and KPC-78 contributed to the high-level CZA resistance observed in the clinical isolates. Previous studies have shown that overexpression of efflux pumps and AmpC enzymes in *P. aeruginosa* can contribute to CZA resistance (23, 24). Therefore, we quantified the relative expression levels of efflux pump genes (*mexA*, *mexC*, *mexE*, and *mexY*) and the AmpC-related gene *bla*_PDC_ to determine whether these factors were associated with CZA resistance in the two isolates (Fig. 4).

Our results showed that *mexD*, *mexE*, and *mexY* were not significantly overexpressed in either strain, whereas *bla*_PDC_ and *mexA* were significantly upregulated in SY-206885 (*bla*_PDC_: 10.766 ± 1.854; *mexA*: 12.142 ± 2.252) and HZ-231016032 (*bla*_PDC_: 7.288 ± 0.852; *mexA*: 13.19 ± 2.26) relative to the susceptible *P. aeruginosa* PAO1 reference strain. Consistent with the selective upregulation of *mexA*, whole-genome sequence analysis identified shared amino acid substitutions in *nalC* (G71E and S209R) and *nalD* (A37T), two known regulators of the MexAB-OprM efflux system, whereas no mutation was detected in *mexR*. These findings suggest that the MexAB-OprM efflux pump and AmpC enzymes may contribute to CZA resistance in these isolates. To further validate this interpretation, we performed efflux pump inhibition assays. In the presence of PaβN, the CZA MIC decreased 32-fold for SY-206885 and 16-fold for HZ-231016032. These results support the conclusion that efflux activity, together with AmpC overexpression, likely contributes to the elevated CZA resistance phenotype of the clinical isolates.

### Structural analysis

**Fig 5:**
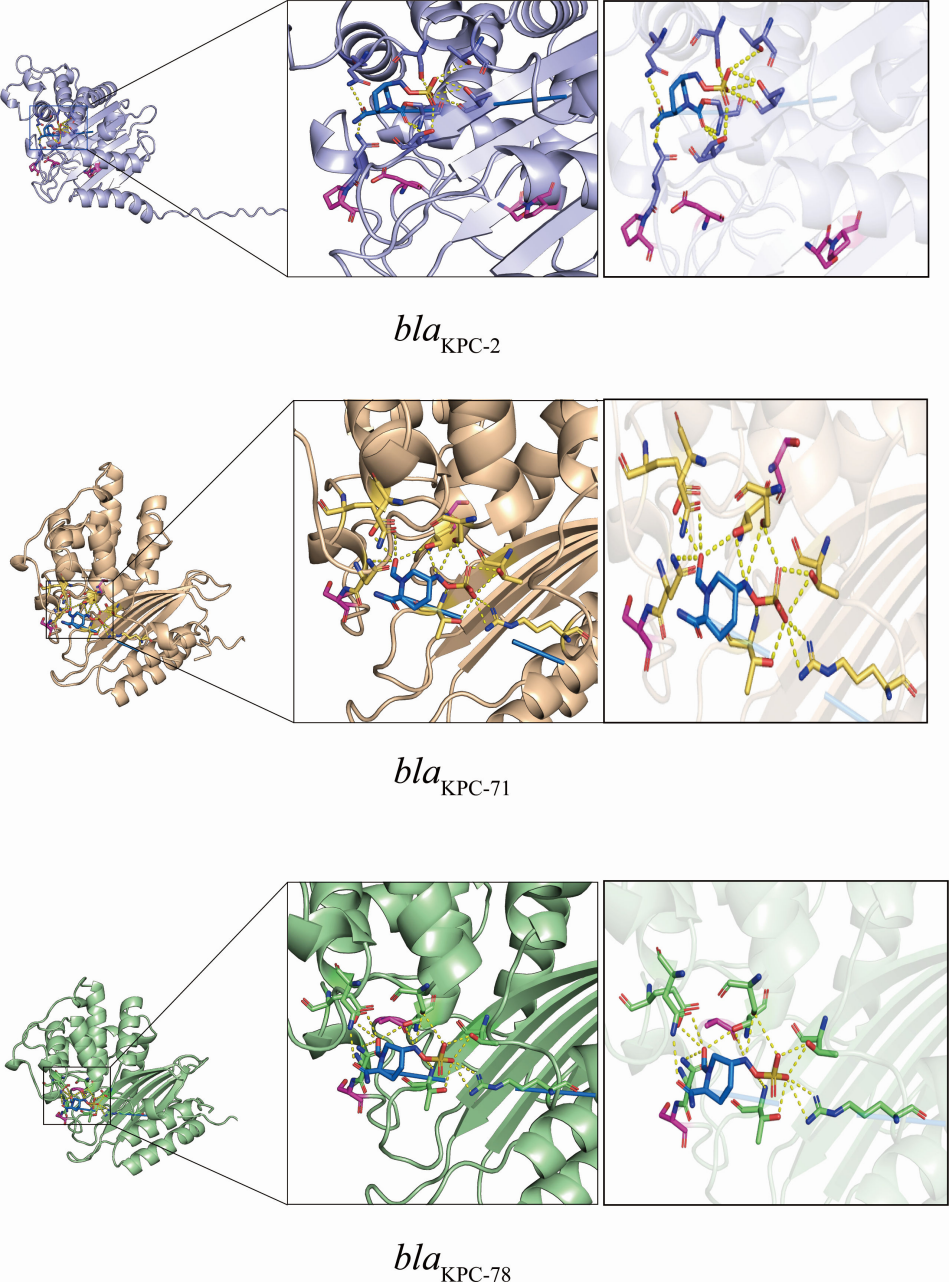
Binding mode analysis of avibactam with different KPC variants, KPC-2, KPC-71, and KPC-78 with their ligands are colored purple, orange, and green, respectively. Avibactam is colored blue.

Homology modeling and covalent docking were performed to assess the ligand-binding properties of KPC-2, KPC-71, and KPC-78. The covalent docking affinity between wild-type KPC-2 and avibactam was −9.2 kcal/mol, whereas the corresponding values for KPC-71 and KPC-78 were −7.3 kcal/mol and −7.4 kcal/mol, respectively, indicating weaker predicted interactions between avibactam and the variant enzymes. These structural observations were consistent with the kinetic data and antimicrobial susceptibility results. Avibactam targets the conserved active-site pocket in which Ser70 acts as the catalytic nucleophile (12). The 182S insertion in KPC-71 and the D179A substitution in KPC-78 are predicted to alter the conformation of the Ω-loop and reshape the active-site environment. These changes may create a more permissive binding pocket for ceftazidime while simultaneously disfavoring productive avibactam engagement (Fig. 5). In the docking models, avibactam adopted a displaced binding pose in both variants, with altered polar interactions involving residues such as Asn170, Ser70, and Glu165. This shift was associated with an increase in binding free energy of approximately 1.9 kcal/mol, consistent with substantially reduced inhibitor affinity. Collectively, these findings suggest that active-site structural alterations weaken avibactam inhibition and thereby contribute to the elevated CZA resistance conferred by KPC-71 and KPC-78.

## DISCUSSION

Ceftazidime-avibactam has become an important therapeutic option for infections caused by KPC-producing gram-negative pathogens, including some CRPA isolates and Enterobacterales (11, 25). However, the increasing clinical reliance on CZA imposes selective pressure that drives the evolution of KPC variants. In this study, we characterized two KPC variants, KPC-71 and KPC-78, emerging from the high-risk ST463 *P. aeruginosa* lineage. Our findings indicate that these structural mutations confer CZA resistance at the cost of reduced carbapenem hydrolytic activity, a phenomenon that poses significant diagnostic and therapeutic challenges.

The primary mechanism of CZA resistance in these variants is attributable to specific amino acid alterations—the 182S insertion in KPC-71 and the D179A substitution in KPC-78. Both alterations affect the region surrounding the Ω-loop, a key structural element that influences active-site architecture and substrate recognition in class A β-lactamases (21, 26). Consistent with previous studies on KPC-33 and KPC-50 (18, 27), mutations in this region destabilize the active site geometry. Our kinetic profiling and molecular modeling suggest that these alterations modify the active-site geometry and perturb the interaction network surrounding Ser70, thereby reducing the efficiency and stability of avibactam-mediated inhibition. This is evidenced by the significantly elevated *K*_*i*_ and IC_50_ values for avibactam. Paradoxically, while these mutations improve ceftazidime accommodation and reduce susceptibility to avibactam inhibition, they severely impair the enzyme’s ability to hydrolyze carbapenems, resulting in the “ceftazidime-avibactam-resistant, carbapenem-susceptible” phenotype observed in our transformants. This “see-saw” effect highlights the plasticity of the KPC enzyme but also warns clinicians that phenotypic susceptibility to carbapenems in these strains may be deceptive, as reversion mutations could theoretically restore carbapenem resistance under imipenem or meropenem pressure.

While the specific KPC mutations are the primary determinants of CZA resistance, our data indicate that intrinsic resistance mechanisms in *P. aeruginosa* play a synergistic role. The clinical isolates SY-206885 and HZ-231016032 exhibited substantially higher CZA MICs (SY-206885: 1024 μg/mL; HZ-231016032: 512 μg/mL) compared to the PAO1 transformants (32 μg/mL). Gene expression analysis revealed significant overexpression of the *mexAB-oprM* efflux system and the chromosomal AmpC (*bla*_PDC_) in the clinical isolates. The increased expression of *mexA* observed in both isolates may be associated with alterations in known MexAB-OprM regulatory genes, since both strains carried substitutions in *nalC* (G71E and S209R) and *nalD* (A37T), whereas no mutation was identified in *mexR*. Previous studies have reported the *nalC* substitutions G71E and S209R in an isolate with elevated *mexA* expression (28); however, their precise contribution to the multidrug-resistant phenotype in *P. aeruginosa* remains unclear. This suggests a multi-layered resistance model: the mutated KPC enzymes provide the specific biochemical barrier against avibactam inhibition, while overexpression of efflux pumps and AmpC further elevates the resistance threshold, leading to the high-level resistance phenotype seen in the clinical setting. Recent mechanistic studies have suggested that overexpression of the MexAB-OprM efflux system contributes to reduced CZA susceptibility in *P. aeruginosa* (29, 30). In this context, efflux pump inhibitors (EPIs) may represent a potential adjunctive strategy, as inhibition of active efflux could increase the intracellular exposure to ceftazidime and enhance the activity of antipseudomonal agents. Therefore, the development and clinical evaluation of safe EPIs in combination with CZA warrant further investigation. Because *bla*_KPC-71_ and *bla*_KPC-78_ were located on native plasmids in the clinical isolates, precise reversion of the mutant alleles to wild-type *bla*_KPC-2_ in the original strain backgrounds was technically difficult. Therefore, although the cloning experiments support a direct contribution of these variants to CZA resistance, the effects of plasmid context and strain-specific background mechanisms cannot be completely excluded.

Epidemiologically, the identification of these variants within the ST463 lineage is alarming. ST463 has emerged as a major high-risk CRPA clone in China and, in some recent studies, has been reported with increasing frequency relative to ST235 (31, 32). Unlike ST235, which is often associated with metallo-β-lactamases, ST463 is typically KPC-producing and has been associated with enhanced virulence potential (33). The localization of *bla*_KPC-71_ and *bla*_KPC-78_ on mobile plasmids containing conserved IS*26*-IS*Kpn27* elements may facilitate horizontal dissemination of these resistance determinants, not only among *P. aeruginosa* populations but potentially to other gram-negative pathogens (34, 35). In addition, the recovery of these isolates from patients with prior exposure to β-lactams or CZA is consistent with selection under antimicrobial pressure during therapy.

In conclusion, the emergence of KPC-71 and KPC-78 in ST463 *P. aeruginosa* represents a significant escalation in the complexity of antimicrobial resistance. The structural modification of the KPC Ω-loop confers resistance to the last-line agent CZA while altering the hydrolytic profile of the enzyme. Clinicians should be vigilant regarding this resistance phenotype, particularly in patients with recent CZA exposure. Furthermore, the combination of mCIM and molecular genotyping remains essential for the accurate detection of these variant KPC enzymes, as standard carbapenem susceptibility testing alone may not fully reflect the underlying resistance mechanism.

### Conclusions

In conclusion, we identified two plasmid-associated KPC variants, KPC-71 and KPC-78, in clinical ST463 *P. aeruginosa* isolates and demonstrated that both variants confer ceftazidime-avibactam resistance while reducing carbapenem hydrolytic activity. This resistance phenotype is linked to structural changes affecting the active-site region and is further enhanced in the clinical isolates by additional mechanisms such as efflux and AmpC overexpression. Given the clinical importance and dissemination potential of the ST463 lineage, continued molecular surveillance is needed to monitor the emergence and spread of CZA-resistant KPC variants during therapy.

## MATERIALS AND METHODS

### Patient and isolate data

*P. aeruginosa* strain SY-206885 was isolated from the sputum of a 74-year-old patient following 11 days of treatment with CZA and ganciclovir for recurrent pulmonary infection; the strain exhibited high-level CZA resistance (MIC >128 mg/L). Strain HZ-231016032 was isolated from a 70-year-old patient with severe pneumonia who had been treated with multiple antimicrobials, including cefepime, amikacin, and CZA. Species identification was performed using matrix-assisted laser desorption ionization–time of flight mass spectrometry (bioMérieux, France) and confirmed by WGS. *P. aeruginosa* PAO1 and its rifampicin-resistant mutant (PAO1-RifR) were used for genetic manipulations.

### Whole-genome sequencing and bioinformatics

The genomic DNA of the *P. aeruginosa* strains SY-206885 and HZ-231016032 was extracted using a QIAamp DNA minikit (Qiagen, Valencia, CA, USA). The raw sequencing read data underwent quality checking via FastQC v.0.11.9 (https://github.com/s-andrews/FastQC), and adapter sequences along with low-quality bases were removed using Trimmomatic v.0.39 (https://github.com/usadellab/Trimmomatic). Hybrid *de novo* assembly of Illumina and Nanopore reads was performed using Unicycler v.0.4.8 to generate complete genome sequences (36). Genome annotation was performed using Bakta v.1.6.0 (37). Serotype (O-antigen) prediction was carried out with the PAst tool (https://github.com/Sandramses/PAst). Comparative genome circle diagrams were generated using BRIG (38). Resistance genes and multilocus sequence types (MLST) were identified using ResFinder 4.1 and MLST 2.1 via the Center for Genomic Epidemiology (39). Single-nucleotide polymorphisms in resistance-associated genes were identified by mapping Illumina reads against the *P. aeruginosa* PAO1 reference genome using Snippy v.4.6.0 (https://github.com/tseemann/snippy).

### Antimicrobial susceptibility testing

Susceptibility testing was performed via broth microdilution according to Clinical and Laboratory Standards Institute (CLSI) guidelines (40). MICs were determined in cation-adjusted Mueller-Hinton (MH) broth using standard inocula. MIC values were interpreted according to CLSI breakpoints, except for colistin, which was interpreted according to EUCAST criteria (41). *P. aeruginosa* ATCC 27853 was used as a quality control strain. All MIC determinations were performed in at least three independent experiments.

### Cloning experiments

The *bla*_KPC-71_ and *bla*_KPC-78_ genes, including their upstream promoter regions, were amplified from SY-206885 and HZ-231016032, respectively. Wild-type *bla*_KPC-2_ was amplified from a reference strain. Purified PCR products were cloned into the pUCP24 vector. Recombinant plasmids were transformed into *P. aeruginosa* PAO1. Transformants were selected on MH agar containing 50 mg/L gentamicin and verified by PCR and Sanger sequencing. Primers are listed in Table 4.

**TABLE 4:** Plasmids construction, conjugate transfer verification, and quantitative PCR primers in this study

Primer name	Primer sequences (5′−3′)
pUCP24-blaKPC F	ACGAATTCGAGCTCGGTACCATGTCACTGTATCGCCGTCT
pUCP24-blaKPC R	CAAGCTTGCATGCCTGCATTACTGCCCGTTGACGC
pET28a-blaKPC F	GCGGATCCGAATTCGAGCTGACCAACCTCGTCGC
pET28a-blaKPC R	CCGCAAGCTTGTCGATTACTGCCCGTTGACGC
Y-pUCP24 F	AGCTGGCACGACAGGTT
Y-pUCP24 R	CTTCGCTATTACGCCAGCTG
Y-pET28a F	TCCGGATATAGTTCCTCCTTTCAGC
Y-pET28a R	GCCATCATCATCATCATCACAGCAG
Y-PAO1-blaKPC F	ATGTCACTGTATCGCCGTCT
Y-PAO1-blaKPC R	TTACTGCCCGTTGACGC
Y-*rpsL* - F	CGGCACTGCGTAAGGTATGC
Y-*rpsL* - R	CGTACTTCGAACGACCCTGCT
Y-*mexA* - F	GGCGACAACGCGGCGAAGG
Y-*mexA* - R	CCTTCTGCTTGACGCCTTCCTGC
Y-*mexD*-F	CGAGCGCTATTCGCTGC
Y-*mexD*-R	GGCAGTTGCACGTCGA
Y-*mexE* - F	TCATCCCACTTCTCCTGGCGCTACC
Y-*mexE* - R	CGTCCCACTCGTTCAGCGGTTGTTCGATG
Y-*mexY*-F	CCGCTACAACGGCTATCCCT
Y-*mexY*-R	AGCGGGATCGACCAGCTTTC

### Conjugation experiments

Conjugation was performed using *P. aeruginosa* SY-206885 and HZ-231016032 as donors and PAO1-RifR as the recipient (42). Transconjugants were selected on MH agar supplemented with 100 μg/mL rifampin and 4 or 8 μg/mL CZA. The presence of *bla*_KPC_ genes was confirmed by PCR and sequencing.

### Carbapenemase detection

Carbapenemase production was assessed using the mCIM according to CLSI guidelines (40), the NG-Test Carba 5 (Gold Mountainriver Tech Development, Beijing, China), and qPCR.

### Protein expression, purification, and enzyme kinetics

To facilitate cytoplasmic expression and purification of soluble KPC enzymes for *in vitro* enzymatic assays, the N-terminal signal peptide sequences (the first 23 amino acids) of *bla*_KPC-71_, *bla*_KPC-78_, and *bla*_KPC-2_ were removed based on previous reports (43). The truncated genes were cloned in-frame downstream of the N-terminal His6 tag of the pET-28a vector. The recombinant plasmids were introduced into *E. coli* BL21 (DE3) by heat shock. Protein expression was induced at mid-logarithmic phase with 0.1 mM isopropyl β-D-1-thiogalactopyranoside and continued at 15°C with shaking at 150 rpm for 20 h. Cells were harvested by centrifugation, lysed, and the recombinant proteins were purified using BeyoGold His-tag Purification Resin (cat. no. P2226) according to the manufacturer’s instructions. Purified proteins were used for subsequent enzymatic assays.

Steady-state kinetic parameters of KPC enzymes toward β-lactam substrates were determined by measuring initial reaction velocities using a microplate reader (Tecan Group Ltd., Switzerland) at room temperature. Substrates were serially diluted in 1× PBS (phosphate-buffered saline, pH 7.2) to generate 10 different concentrations. Initial velocities were calculated from the linear change in absorbance over time using the corresponding substrate-specific extinction coefficients (ε). The kinetic parameters *K*_*m*_ and *V*_max_ were obtained by fitting the initial velocity data to the Michaelis-Menten equation using nonlinear regression in GraphPad Prism v.10.1.2. *K*_*m*_ represents the substrate concentration at which the reaction rate reaches half of *V*_max_ and reflects the affinity of the enzyme for the substrate. The catalytic turnover number (*K*_cat_) was calculated according to the equation:


$$K_{cat}=\frac{V_{max}}{\left[E\right]}$$

where [*E*] denotes the molar concentration of active enzyme.

For inhibition assays, a fixed concentration of KPC enzyme was preincubated with increasing concentrations of inhibitor [I], followed by the addition of nitrocefin at a final concentration of 100 μM as the substrate. Initial reaction velocities (V) were measured under the same conditions described above. The inhibition constant (*K_i_*), reflecting the binding affinity between the inhibitor and the enzyme, was determined by fitting the velocity data to a competitive inhibition model using GraphPad Prism v.10.1.2, according to the following equation:


$$V=\frac{V_{max}\cdot [S]}{K_{m}\left(1+\frac{\left[I\right]}{K_{i}}\right)+[S]}$$

Lower *K_i_* values indicate stronger inhibitory potency.

Using nitrocefin as the substrate, the inhibition IC₅₀ values of avibactam, tazobactam, and clavulanic acid against the wild-type KPC-2, KPC-71, and KPC-78 were determined. The enzymes were mixed with these inhibitors in PBS at concentrations ranging from 0 to 100 mM, incubated for 10 minutes, and then ceftazidime was added at a concentration of 100 mM. After 30 minutes, the absorbance was recorded at 482 nm and exported to Prism software, where the IC₅₀ values were calculated using the dose-response inhibition, variable slope (four-parameter) equation.

### Efflux pump inhibition

The MICs of CZA were determined in the presence and absence of PAβN (TaKaRa Bio Inc., Otsu, Shiga, Japan) at a concentration of 50 mg/mL (44). The isolates were confirmed to overexpress efflux pumps when the MICs in the presence of PAβN were determined to be at least fourfold lower than the MICs in the absence of PAβN (44). The wild-type *P. aeruginosa* PAO1 strain was used as the reference strain.

### Structure modeling and molecular docking

Homology models for KPC-71 and KPC-78 were built using the Prime module in Schrödinger, based on the KPC-2 crystal structure (PDB: 3DW0). Structures were prepared using the Protein Preparation Wizard with OPLS3e force field minimization (45, 46). Binding affinities were estimated using Prime MM/GBSA scoring (47, 48).

## Data Availability

The complete genome sequences of P. aeruginosa strain SY-206885 and HZ-231016032 strains in this study are deposited in Figshare (https://doi.org/10.6084/m9.figshare.31167700).
